# An Experimental Study of Briquetting Process of Torrefied Rubber Seed Kernel and Palm Oil Shell

**DOI:** 10.1155/2016/1679734

**Published:** 2016-06-21

**Authors:** M. Fadzli Hamid, M. Yusof Idroas, M. Zulfikar Ishak, Z. Alimuddin Zainal Alauddin, M. Azman Miskam, M. Khalil Abdullah

**Affiliations:** ^1^School of Mechanical Engineering, Universiti Sains Malaysia, Engineering Campus, Seri Ampangan, 14300 Nibong Tebal, Pulau Pinang, Malaysia; ^2^Science and Engineering Research Centre, Universiti Sains Malaysia, Engineering Campus, Seri Ampangan, 14300 Nibong Tebal, Pulau Pinang, Malaysia; ^3^School of Materials and Mineral Resources Engineering, Universiti Sains Malaysia, Engineering Campus, Seri Ampangan, 14300 Nibong Tebal, Pulau Pinang, Malaysia

## Abstract

Torrefaction process of biomass material is essential in converting them into biofuel with improved calorific value and physical strength. However, the production of torrefied biomass is loose, powdery, and nonuniform. One method of upgrading this material to improve their handling and combustion properties is by densification into briquettes of higher density than the original bulk density of the material. The effects of critical parameters of briquetting process that includes the type of biomass material used for torrefaction and briquetting, densification temperature, and composition of binder for torrefied biomass are studied and characterized. Starch is used as a binder in the study. The results showed that the briquette of torrefied rubber seed kernel (RSK) is better than torrefied palm oil shell (POS) in both calorific value and compressive strength. The best quality of briquettes is yielded from torrefied RSK at the ambient temperature of briquetting process with the composition of 60% water and 5% binder. The maximum compressive load for the briquettes of torrefied RSK is 141 N and the calorific value is 16 MJ/kg. Based on the economic evaluation analysis, the return of investment (ROI) for the mass production of both RSK and POS briquettes is estimated in 2-year period and the annual profit after payback was approximately 107,428.6 USD.

## 1. Introduction

The strategy of torrefaction of bulky biomass material for combustion product has been highlighted in the last decades in order to replace fossil fuel as the primary energy [1]. It provides the lowest greenhouse gas alternative and is being studied by many countries. Torrefaction process is a process of converting an organic substance into carbon-containing residue through heating or destructive distillation [2]. It is a thermal process by which biomass is treated in an inert atmosphere at a temperature of 227–677°C. Torrefaction process enhances the physical characteristics of biomass by having more homogeneous composition, high energy density, low moisture content, and hydrophobic behavior. These added values of torrefied biomass provide a very good market and help to improve the overall economics of the biomass utilization process for energy production. However, the production of this torrefied biomass is loose, powdery, and nonuniform. One method of upgrading this material in improving their handling and combustion properties is by densification into briquettes of higher density than the original bulk density of the material. Densification is capable of increasing the density of the biomass feedstock at approximately 66%. It will simplify the uniform shape and size, facilitates the handling and storage, and easily is adopted in direct combustion [1, 3–5]. In the densification process, the techniques involved are via either mechanical densification or pyrolysis. The mechanical densification technique usually involves the application of pressure to densify the material. The pyrolysis technique usually involves preheating the biomass in absence of oxygen. Mechanical densification involves six popular techniques, that is, bales, pellets, cubes, briquette, wood chips, and pucks. In pyrolysis densification, there are three common popular techniques (i.e., torrefaction, slow pyrolysis, and fast pyrolysis). Nevertheless, pyrolysis is more expensive to densify compared to the mechanical densification such as cubes, pucks, briquette, and wood chips, which are more feasible in terms of the quantity produced and less expensive. The factors that affect the cost of densification are classified as its raw material, equipment, and personal costs, as well as operating time (hours/day) and size of densification plant (tonnes/year) [6]. The properties for any biofuel consist of its physical and chemical properties which include density, moisture content, heating value, ash content, and also its mechanical properties such as impact and compressive strength, as well as handling and storage. The briquettes have many advantages over standard torrefied biomass that includes a complete dryness and dense of briquettes that leads to an inexpensive shipment and storage, no water absorption of the briquettes for an outdoor storage and shipment, and a comparable heating value to coal and biomass briquettes that require no modifications to the existing coal-fired power plant.

System variable controlling the densification is a vital stage in order to achieve the desired density, durability, and improved quality. The quality of the briquette depends on the number of process variables such as temperature, pressure, usage of binder, preheating of biomass mixture, use of additives, and change of blend formulation [7]. The compaction of biomass during the briquetting process is attributed by two conditions, which are elastic condition and plastic deformation [8, 9]. Smooth briquetting refers to an improvement in the productivity rate with a minimum process downtime due to material clogging in the screw extrusion section. According to Tabil Jr. [10], there are two important aspects to be considered in the compaction of biomass material, which are the ability of particles to form briquettes with extensive mechanical strength and the ability of process to enhance the durability of the biomass material. In order to achieve a better densification, type of bonding and mechanical interlocking are the fundamental issues to be dealt with in biomass material compaction. The presence of liquid-like water as a binder during briquetting is the current factor that attracts the attention of many researchers to perform further investigation on biomass densification. It has been discovered that the presence of liquid enhances the interfacial forces and capillary pressure and increases the particle bonding. The attraction between particles is proportional to the Van der Wall's electrostatic or magnetic force. The attraction relies on the distance between the particles where the furthest distance has less attraction. Mani et al. [11] studied and concluded that there are three critical stages during densification of biomass. The first stage is the rearrangement of particles to reform a closely packed mass and the dissipation of energy due to interparticle and particle wall frictions. The second stage is that the particles are pushed against each other and undergo plastic and elastic deformation, which increases the interparticle contact significantly. The particles become bonded through Van der Wall's electrostatic forces. Finally, for the third stage, a significant decrease in volume at higher pressure results in the compactness of the briquettes reaching the factual density of the constituent elements.

This paper presents the technology of converting biomass into biofuel material with improved calorific value via torrefaction process that was successfully developed and tested at the Bio-Energy Laboratory, School of Mechanical Engineering, Universiti Sains Malaysia (USM). The salient features of USM torrefaction system comprise a continuous and steady thermochemical conversion process via screw feeding/extrusion principle resulting in the increase of biofuel production rate by 400% per hour (200 kg/hr) as compared with the batch type (50 kg/hr). The design of the USM system that incorporates the optimum design layout, fuel burner, and screw feeding/extrusion permits a steady regulation and control of carbonization temperature and residence time. These advantages will reduce both manufacturing and operation costs significantly as far as the mass production of biofuel is concerned. The salient features of USM thermochemical conversion system can definitely benefit the company that works with thermochemical conversion of biomass and/or agricultural wastes to biofuel in mass production. Furthermore, the purpose of this study was to investigate the quality of densified biomass materials, which are rubber seed kernel (RSK) and palm oil shell (POS) in order to optimize the best composition to enhance the compressive strength and calorific value, respectively.

## 2. Methodology

### 2.1. Raw Biomass Materials and Torrefaction Process

**Figure 1:**
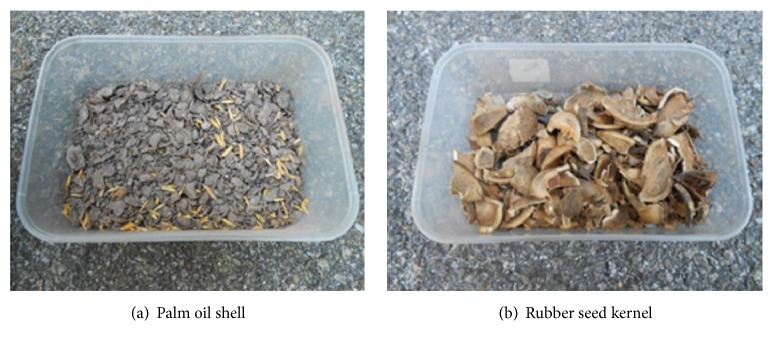
Samples of raw biomass materials.

The raw biomass materials used were rubber seed kernel (RSK) and palm oil shell (POS) due to their wide availability in Malaysia [3], with a considerable amount of calorific value (CV) of 16 MJ/kg and 17 MJ/kg, respectively. Their physical and combustion properties were determined via standard compressive load and bomb calorimeter tests.  Figure 1 shows the raw POS and RSK samples prior to the torrefaction process.

**Figure 2:**
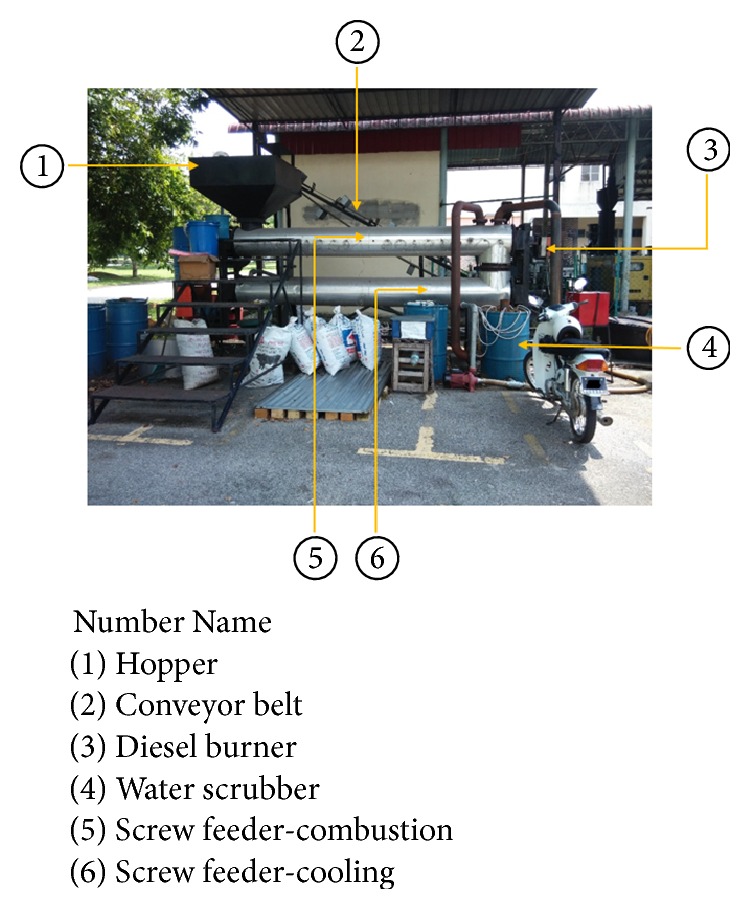
USM continuous torrefaction system.

For energy production of torrefied biomass, the raw biomass materials were torrefied and then ground into smaller particles.  Figure 2 shows the continuous torrefaction system to torrefy the raw biomass materials using the heated screw extruder.

**Figure 3:**
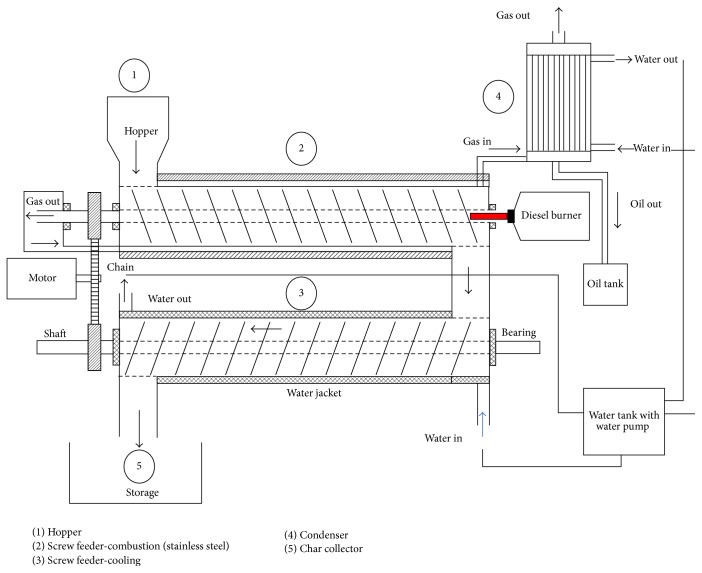
USM continuous thermochemical conversion system diagram.

Figure 3 shows the USM continuous thermochemical conversion system diagram. The system operated such that the biomass and/or agriculture waste material were fed into the hopper. The diesel burner or biomass gas burner (can be operated with the oil and gas fuel of the products of the process) was used to heat up the screw conveying heating unit. The perfect control of temperature of this screw heating unit allowed the treating of a very wide range of biomass fuel and gave possibilities to vary production of biooil or biochar depending on the treated biomass fuel. The furnace was developed in a double jacket to allow heated gas from the diesel burner to be pumped through and circulated for the better thermal distribution. The proper insulation outside the furnace was also developed for the same reason and for safety factors. The screw heating unit conveyed the biomass fuel along the furnace (at about 200–800°C) which was set to a rotating state of 1 to 2 rpm for mixing, homogenization, reactions, and heating of the material for the complete pyrolysis reaction to take place uniformly and continuously during 3 to 6 hours of the reactions and heating processes. The cooling system was also based on screw conveyor in a double jacket with a coolant water circulation inside. This system allowed stocking of the biochar from the heating unit outlet directly in bags or other containers. At the end of the heating furnace, a heat exchanger collected the gas from the heated biomass to condense them in two phases; one was called producer gas which is a noncondensable gas and another one which is biooil. The biooil was collected in an oil tank while the producer gas/syngas was fed up to the gas burner for heating the furnace element. The outlet flue gases of the burner were mainly carbon dioxide which could be fed into carbon dioxide pilot plant where it was purified and compressed into liquid or solid carbon dioxide. This continuous production process of biooil, biochar, and gas fuel was an innovative process developed for thermochemical conversion of biomass and waste. The most salient feature of the continuous system was its capability to produce the biomass output at 200 kg/hr compared to 50 kg/hr for the conventional batch-type system, resulting in the productivity improvement by 400%.

**Figure 4:**
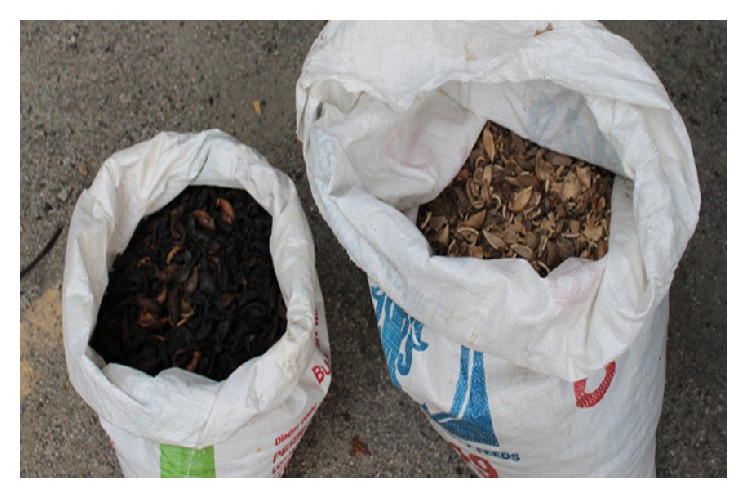
Sample torrefied biomass (left) in comparison with raw biomass (right).

Most of the torrefied biomasses were produced in dissimilar shapes and sizes. Thus, they needed to be crushed into small pieces. These particle sizes for both POS and RSK were approximately in between 15 *μ*m to 90 *μ*m. The crushing of both torrefied POS and RSK made them pulverized in smaller sizes (less than 1 mm). The steps were crucial in order to make the torrefied biomass dry and easy to be briquetted. The POS and RSK had been significantly heated and dried throughout the torrefaction process. The water contained in the feedstock as well as superfluous volatiles was released, and the biopolymers (cellulose, hemicelluloses, and partly decomposed lignin) gave off various types of volatiles. The chemical properties of biomass improved after the torrefaction process in terms of fuel quality for gasification and/or combustion. The torrefaction process produced a remaining solid, dried, blackened torrefied biomass as shown in Figure 4.

**Figure 5:**
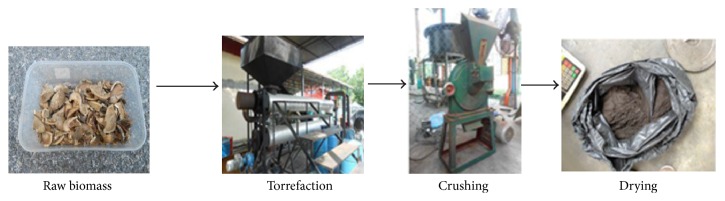
A process flow of raw to torrefied biomass preparation for briquetting.

Figure 5 depicts a complete process flow of raw biomass material undergoing torrefaction, crushing, and drying processes prior to briquetting. The drying process could be done using a dryer machine or by natural drying under the sunlight. For simplicity and low cost factor, the torrefied POS and RSK were dried under the sunlight continuously until the moisture content of the torrefied biomass became less than 12%.

### 2.2. Mixing Process

Prior to briquetting process, the torrefied POS and RSK were mixed with certain compositions of starch as binder addition and water. The percentage composition of the binder addition and water was characterized based on the weight of torrefied biomass used for a smooth briquetting process [12].

The starch and water were weighed according to the desired percentage of composition. Then, they were mechanically mixed and heated for 5–10 minutes until they became sticky. The gluey binder was then mixed with 1 kg of torrefied POS for another few minutes until they were well-mixed [13]. Similar mixing process was applied for torrefied RSK.

### 2.3. Briquetting Process

**Figure 6:**
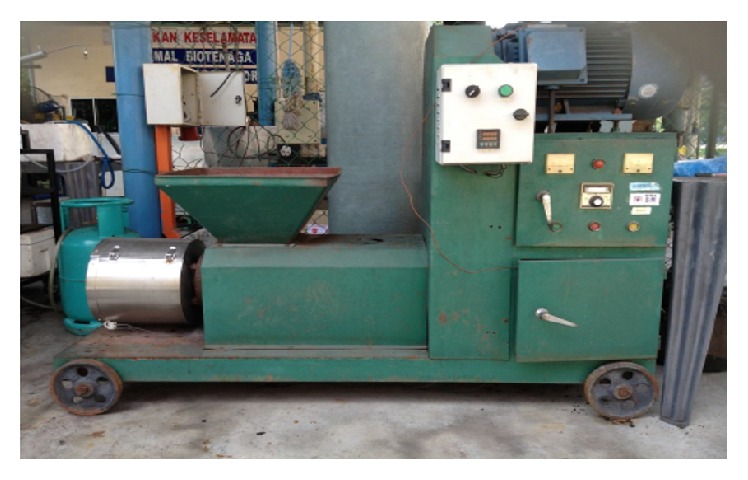
The horizontal type briquette machine.

**Figure 7:**
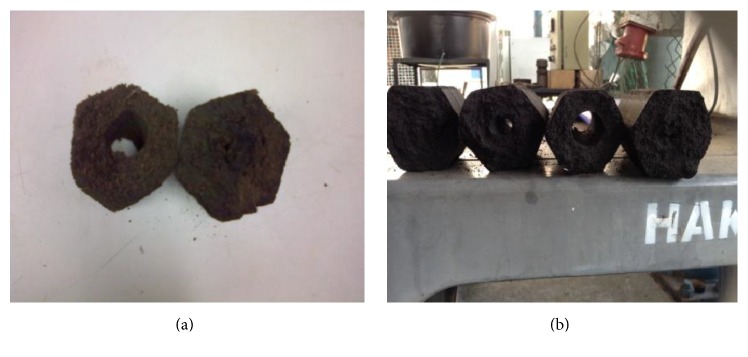
POS (a) and RSK (b) products of briquette.

The well-mixed torrefied POS and RSK with binder were fed into the briquette machine for briquetting. The briquette machine used was the horizontal type with screw extruder and heater as shown in Figure 6. This briquette machine has been extensively used to briquette raw biomass materials such as palm oil shell and wood sawdust [13]. The use of heater band in the screw extrusion section was to heat up the torrefied biomass at the operating temperature of 100°C to 500°C in order to aid in building up the pressure and to allow a smooth exit of the briquettes. Consequently, it improved the productivity rate to match the required capacity of the continuous torrefaction system at 200 kg/hr.  Figure 7 shows the briquette products of POS and RSK in the hexagonal shape with the size of 5 cm high and 2 cm for inner diameter.

### 2.4. Compressive Load Test

The compressive load test was performed to determine the maximum compressive load that the biomass briquette could withstand before cracking. The compressive load test was attributed to predetermine the elastic and plastic deformation of the densified briquette strength composition. The compressive load test machine used in this experiment was Model INSTRON 3367. The speed of the moving platform was set at 5 mm/min. The program was set to increase the load applied on the scale of 0.01 N. The briquette was placed horizontally on the fixed platform of the machine and the moving upper platform was set to be in contact with the briquette and further compressed until deformation or cracking occurred.

### 2.5. Bomb Calorimeter Test

The calorific value of biomass briquette as the fuel sample was determined using a Nenken Type Adiabatic Bomb Calorimeter. The mass of paper and the mass of biomass sample were measured. The solid biomass sample was wrapped with a rice paper. A nichrome wire length was measured approximately 1 cm and tied together with the solid fuel. The sample was placed in a crucible and put into the vessel and the bomb to ignite and measure its energy value. The vessel was filled with oxygen, approximately 30 bars, and placed inside the calorimeter. The vessel was surrounded by water (insulation) and the water circulation was realized by mechanical agitation via rotation of the blades. The temperature was measured in parallel with the time taken until no more energy rise.

The calorific value was calculated by the following equation [14]:

(1) $$\begin{array}{c} CV=\frac{\left\{M_{cw}+M_{wic}\right\}\times T_{corr}\times {c_{p}}_{w}-\left(E_{rp}+E_{nw}\right)}{M_{s}}, \end{array}$$

where *M*_cw_ is the equivalent water mass of the calorimeter, *M*_wic_ is the mass of water, *T*_corr_ is the corrective temperature, *c*_*p*__w_ is the specific heat capacity of water, *E*_rp_ is the energy of rice paper, *E*_nw_ is the energy of nickel wire, and *M*_s_ is the mass of sample.

### 2.6. Scanning Electron Microscope (SEM)

The microstructural analysis of torrefied POS and RSK briquettes was conducted using SEM method. The mechanical structure relating to mechanical strength of the torrefied biomass briquettes was determined via morphological analysis. The area surface topography of the torrefied POS and RSK briquettes and their quality of solidification substance manner were determined.

### 2.7. Economic Evaluation of Biomass Briquetting

The financial cost of biomass briquetting process is very much depending on the types of biomass material used and their material handling [15]. This section presents the estimation costs that comprises of both capital and operational costs.

Capital cost is considered as a one-time expense to purchase equipment, land, transportation, and facilities. The capital cost refers to the needs of expenditure in order to bring the project to a commercialization operation status. The total capital cost was calculated by the following equation [15].

Total capital cost, *C*_c_, is as follows:

(2) $$\begin{array}{c} C_{c}=eC_{eq}, \end{array}$$

where *C*_eq_ is the cost of equipment and *e* is the capital recovery factor. A capital recovery factor is the function of converting the present value into a stream of equal annual payment over a specified period of time. The capital recovery factor was calculated by the following equation [15].

Capital recovery factor, *e*, is as follows:

(3) $$\begin{array}{c} e=\frac{i{\left(1+i\right)}^{N}}{{\left(1+i\right)}^{N}-1}, \end{array}$$

where *i* is the interest rate and *N* is the lifetime of the equipment in years [15].

The equipment cost, *C*_eq_, is as follows:

(4) $$\begin{array}{c} {C_{eq}=\alpha_{eq}p}^{n_{eq}}, \end{array}$$

where *C*_eq_ is the unit cost of equipment, *n*_eq_ is the scaling factor of equipment, and *p* is the characteristic parameter of equipment [15].

The Return on Investment Formula is as follows:

(5) $$\begin{array}{c} \text{ROI}=\frac{\text{Gain from investment}-\text{Cost of Investment}}{\text{Cost of Investment}}, \end{array}$$

where Gain from investment refers to profits obtained from the sale of investment while Cost of investment refers to the initial cost to invest for system development.

## 3. Results and Discussion

The torrefied biomass materials produced were dry and brittle in characteristics and this has provided a significant advantage to crushing and briquetting of the torrefied biomass as compared to raw biomass. The briquetting of torrefied POS and RSK has been established based on the characterization process of mixing them at certain compositions of binder addition (% S) and water (% W). The briquetting of these materials has been successfully conducted at the maximum ambient operating temperature of 100°C since the briquetting at more than 100°C has resulted in material degradation during extrusion and the exit of torrefied POS and RSK briquettes (not in a proper shape) at higher temperature is quite hazardous. Specifically, for torrefied POS and RSK, the best quality of the briquette was produced at the ambient temperature of the briquetting process.

### 3.1. Maximum Compressive Load (MCL)

**Figure 8:**
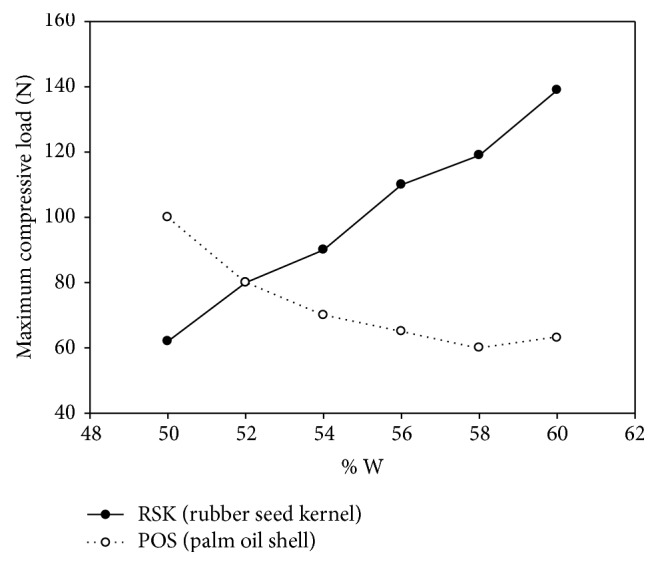
MCL as a function of varying water (constant 5% binder addition).

Figure 8 shows the variations of MCL of both torrefied RSK and POS briquettes with different compositions of water at the constant 5% binder addition. It is shown that the highest MCL for torrefied RSK briquette is 141.36 N at 60% W and the lowest load of 62.62 N at 50% W, while the highest MCL for torrefied POS briquette is 101.11 N at 50% W and the lowest MCL of 57.07 N at 58% W. The curve trends show that the MCL for torrefied RSK increased with the increase of water unlike for torrefied POS. Nevertheless, the torrefied POS has increased slightly at 60% W composition at approximately 4.9% of MCL. This result is in agreement with Mani et al. [16] who indicated that the increase of water composition percentage in the biomass during densification process would act as a binder to improve the bonding via Van Der Waal's forces and increase the contact area of the particles. The test result was valid at the maximum of 60% W of water composition since the mixture of more than that has resulted in liquefaction of the mixture and is inappropriate for briquetting process. In addition, the structure of the torrefied RSK briquette was found to be more stable and stronger than the torrefied POS briquette because the capillary and liquid state in the POS consisted of voids in macroscopic size like a ring at the point of contact between boundaries [17]. The size of voids has a significant influence on the bonding strength characteristic of the biomass and it depends on the negativity of the capillary pressure and surface tension of the liquid [18, 19]. Thus, the combination of the binder hardening, solidification of the melted substance, and a proper pressure applied to the densification is almost stimulus to the mechanism of binding characteristic [19]. The torrefied RSK briquette has a vigorous expansion in the range of 50% to 60% of water composition due to a good adhesion and the gluey characteristic of the mixture that improves the bonding and densification during the briquetting process.

**Figure 9:**
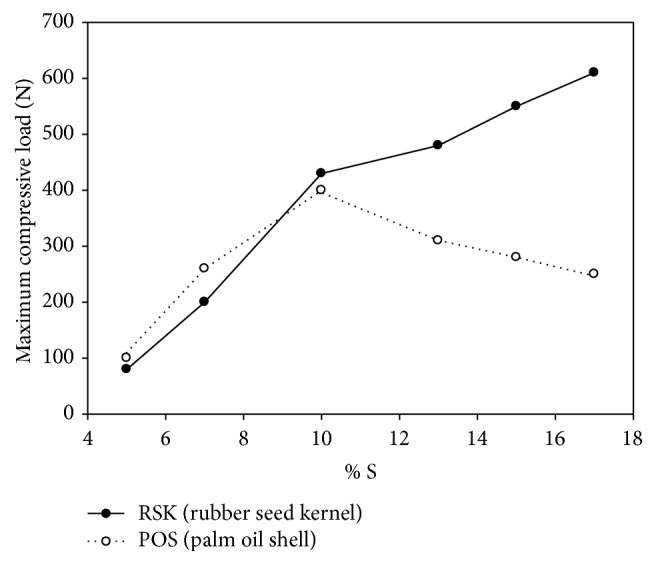
MCL as a function of varying binder addition (constant 50% of water).

The variations in MCL of torrefied RSK and POS briquettes with different percentage of binder addition at 50% constant of water are shown in Figure 9. The result shows that the highest MCL for torrefied RSK briquette is 615.15 N at 17% S and the lowest MCL of 68.63 N at 5% S. However, the highest MCL for torrefied POS briquette is 450.09 N at 10% S and the lowest MCL is 101.11 N at 5% S. The curve trends show that there is a significant improvement in the MCL of both briquettes at the increasing trend of the binder addition until 10%. However, the trend of torrefied RSK briquette after 10% mixture increased but the trend for torrefied POS briquette decreased almost 38%. The increase of MCL was due to the improvement of the adhesive and gluey characteristic of the mixture that further improved the concentration, bonding, and densification of the torrefied biomass. The appropriate composition of starch for torrefied POS briquette was limited to 10% of binder addition. Amerah [20] discovered that the binding/adhesion characteristic of biomass depends more on the amylose to amylopectin ratio of starch. Amylose and amylopectin are two families of homopolysaccharides constituting starch. During their biosynthesis within starch granules, amylose forms double helices immediately that may aggregate (hydrogen bonds) to each other and create semicrystallines region [21]. From the aspect of the briquetting process, the composition of starch has been controlled at the maximum of 17% binder addition. The excesses of the binder addition resulted as a material clogging problem in the screw extrusion, which increased the wearing of the parts and required frequent maintenance.

### 3.2. Calorific Value (CV)

**Figure 10:**
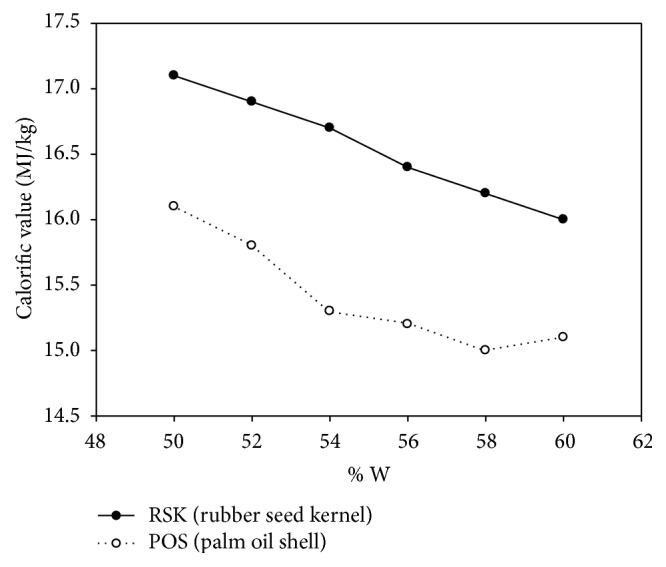
CV as a function of varying water (constant 5% binder addition).

Figure 10 shows the variation of CV of torrefied RSK and POS briquette as a function of varying water at the constant of 5% binder addition. The result shows that the highest calorific value for RSK is 17.07 MJ/kg at 50% W and the lowest is 16.03 MJ/kg at 60% W, while the highest composition for POS is 16.05 MJ/kg at 50% W and the lowest is 15 MJ/kg at 60% W. The trends show that the increasing water percentage composition lowered the CV. Thus, whenever water content was increased, the amount of the RSK and POS would decrease and the water which replaced that volume had no energy to burn the fuel which lowered the CVs for RSK and POS. However, the difference in CVs between RSK and POS is approximately 6.5% of total average difference and this is subjected to the effect of briquetting process conditions such as temperature, particle sizes, pressure, and in-feed pretreatment [22].

**Figure 11:**
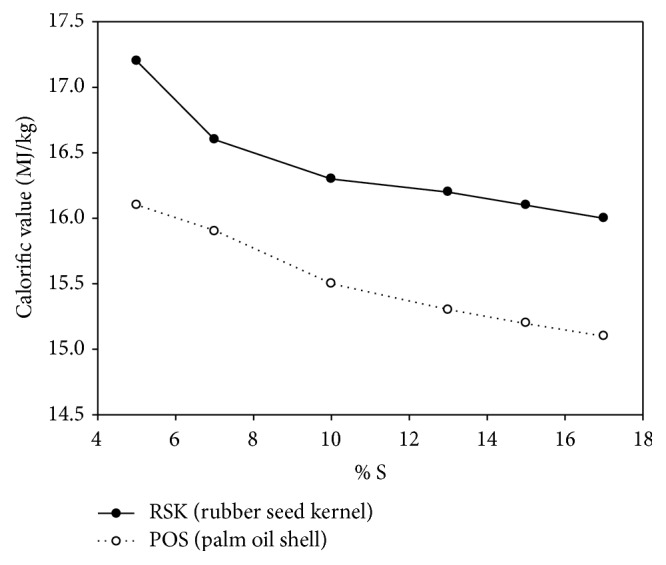
CV as a function of binder addition (constant 50% of water).

Figure 11 shows the variation in CV of torrefied RSK and POS briquette as a function of binder addition at the constant of 50% of water. The result shows that the highest CV for RSK is 17.07 MJ/kg at 5% S and the lowest is 16.00 MJ/kg at 17% S while the highest composition for POS is 16.05 MJ/kg at 5% S and the lowest is 15 MJ/kg at 17% S. The trends show that the increasing starch percentage will reduce and degrade the CV of briquetting. The result indicates that the lesser the binder addition in the biomass, the higher the CV that was produced. Ellis et al. [23] discovered that the binder composition of starch granules may consist of nonstarch components such as lipids, protein, and phosphate group. Its behavior is controlled via the gelatinization process at high processing temperatures. The reduction of calorific value at the increase of starch could be influenced by the gelatinization process. Gelatinization of starch is an irreversible process and mainly influenced by the densification process [24] such as residence time, shear effect, water, and heat [25]. The texture of the gelatinized material is influenced by the starch granules reacting at the higher temperature accompanied by moisture content.

### 3.3. Microstructural Analysis of Raw and Torrefied RSK Briquettes

**Figure 12:**
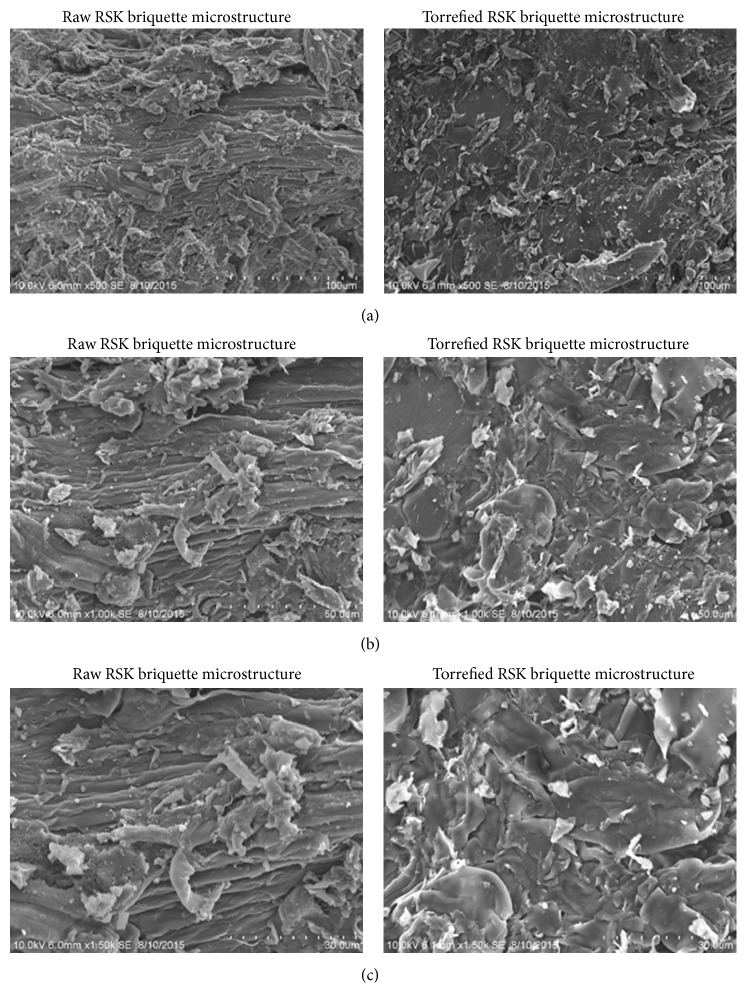
The electron micrographs of raw and torrefied RSK briquettes at the magnification of (a) 100 *μ*m, (b) 50 *μ*m, and (c) 30 *μ*m.

Figure 12 depicts the microstructural analysis of the prior torrefied raw and torrefied RSK briquettes at the magnification of 100 *μ*m, 50 *μ*m, and 30 *μ*m, respectively. The specific torrefaction and briquetting conditions of 60% water and 5% binder were used for the SEM analysis. Based on the result, it was found that the microstructure of torrefied RSK briquette is apparently in fine texture and less porous. This microstructure proves that a good bonding of fine particles and less porosity were observed on torrefied RSK briquette as compared to raw RSK briquette.

### 3.4. Microstructural Analysis of Raw and Torrefied POS Briquettes

**Figure 13:**
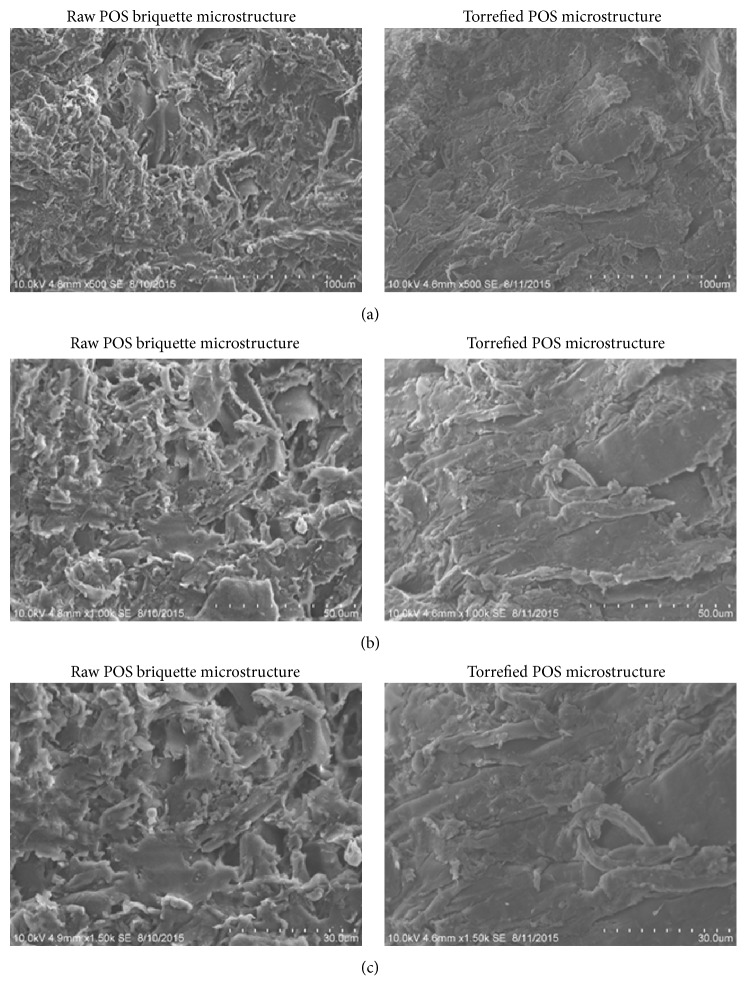
The electron micrographs of raw and torrefied POS briquettes at the magnification of (a) 100 *μ*m, (b) 50 *μ*m, and (c) 30 *μ*m.

Figure 13 also shows the microstructural analysis of both raw and torrefied POS briquettes at the magnification of 100 *μ*m, 50 *μ*m, and 30 *μ*m, respectively. The specific torrefaction and briquetting condition of 60% water and 5% binder were used for the SEM analysis. POS briquette has higher inherent porosity due to its fibrous nature particularly after pulverization. The raw of POS briquette is highly porous and very rich in fine particles. The microstructure of POS is similar to pigmentation and porous structure and much of groove hole in the underneath surface area and very rich in grain particles.

### 3.5. Economic Evaluation Analysis

The USM briquetting machine was experimentally tested for its capability to cope with the continuous torrefaction system at a production capacity of 0.25 t of briquette/h with the annual production of 807 t. The machine is capable of operating 12 h for 269 days annually (annual utilization period 74%). As compared to the conventional systems in the local market such as batch-typed and split system, each thermochemical conversion process has a production capacity of 0.05 t of briquetting/h with the annual production of 322.8 t, where the total improvement almost 60% between the USM briquetting process and the conventional system in terms of annual production output.  Table 1 lists the cost of the equipment purchased with respect to the expected life and the cost is in $/t of pellets produced for each equipment. The transportation cost of raw material to the briquetting operation facility is included. The location of plant is 4 km to the biomass sources. The costs of briquetting machine and miscellaneous equipment are the largest among the annual capital cost.  Table 2 shows the production of biomass briquettes including the variable cost of operation in daily output of 3 tonnes for both RSK and POS. The cost of raw biomass material is among the highest production cost of biomass briquette. The selling price in the market per tonne is 240 USD for RSK and 235 USD for POS. The USM briquetting machine was capable of producing 3 tonnes per day for both RSK and POS in parallel. The net profit per day was estimated as 720 USD for RSK and 705 USD for POS. The annual processing cost for 1 year was 107,428.6 USD. Thus, with reference to the net profit, the return of investment (ROI) was approximately in 2-year period.

## 4. Conclusion

There was a significant effect of optimizing the composition of starch as binder and water to the physical characteristics of the biomass briquettes. In fact, the stronger and more stable particles of the biomass briquettes that improved their hardness and durability was realized by adding the starch as the binder, which controlled its composition together with the composition of water in the mixture prior to the briquetting process. For the POS briquette, the best quality produced was in the torrefied form at the starch composition of 5% S and water composition of 50% W. The maximum compressive load of the POS briquette was 101.11 N and the calorific value was 16.05 MJ/kg. For the RSK briquette, the best quality produced was also in the form of torrefied at the starch composition of 5% S and water composition of 60% W. The maximum compressive load of the RSK briquette was 141 N and the calorific value was 16.03 MJ/kg. Apparently, the RSK briquette is better in terms of the mechanical strength and calorific value than the POS briquette. Further investigations need be conducted on the effect of temperature and pressure on the productivity of briquettes using heater band. It is expected that the activation of lignin and change in the cellulosic structure at the increased temperature and pressure in the briquette machine will aid in the formation of an improved bond and durable briquettes. From the economic evaluation analysis, the return of investment for the mass production of both RSK and POS briquettes was estimated to be in 2-year period with the annual profit of 107,428.6 USD.

## Figures and Tables

**Table 1:** Setup cost of torrefaction plant.

Equipment	Purchase cost ($)	Installation cost ($)	Expected life (y)	Capital recovery factor	Annual capital cost ($)	Specific capital cost ($/t)
Briquetting machine	1000	180	12	0.1254	145	17.9
Storage bin	30	16	20	0.2165	10	1.24
Miscellaneous equipment	200	60	12	0.1254	33	4.08
Screen shaker	30	17	12	0.1254	6	0.74
Land use	40	—	25	0.3033	12	1.48
Office building	80	—	20	0.2165	17	2.10
Front end loader	100	—	12	0.1254	13	1.61
Packaging unit	90	20	12	0.1254	14	1.73

Total	1570	293			250	30.88

**Table 2:** Cost of operation.

Cost of processing/raw material	Rubber seed kernel (250 kg/hr)	Palm oil shell (250 kg/hr)
Capital	90.9	90.2
Diesel	71.4	71.4
Electrical	47.6	47.6
Operator	28.5	28.5

Total cost (USD)	240	237.7
